# Quantitative investigation of memory recall performance of a computational microcircuit model of the hippocampus

**DOI:** 10.1186/s40708-021-00131-7

**Published:** 2021-05-08

**Authors:** Nikolaos Andreakos, Shigang Yue, Vassilis Cutsuridis

**Affiliations:** 1 School of Computer Science, University of Lincoln, Brayford Pool, Lincoln, LN6 7TS UK; 2 Lincoln Sleep Research Center, University of Lincoln, Lincoln, LN6 7TS UK

**Keywords:** Computer model, Dendrite, Inhibition, Excitation, Bistratified cell, Medial septum, Pyramidal cell, Theta rhythm, Memory retrieval, OLM cell

## Abstract

Memory, the process of encoding, storing, and maintaining information over time to influence future actions, is very important in our lives. Losing it, it comes with a great cost. Deciphering the biophysical mechanisms leading to recall improvement should thus be of outmost importance. In this study, we embarked on the quest to improve computationally the recall performance of a bio-inspired microcircuit model of the mammalian hippocampus, a brain region responsible for the storage and recall of short-term declarative memories. The model consisted of excitatory and inhibitory cells. The cell properties followed closely what is currently known from the experimental neurosciences. Cells’ firing was timed to a theta oscillation paced by two distinct neuronal populations exhibiting highly regular bursting activity, one tightly coupled to the trough and the other to the peak of theta. An excitatory input provided to excitatory cells context and timing information for retrieval of previously stored memory patterns. Inhibition to excitatory cells acted as a non-specific global threshold machine that removed spurious activity during recall. To systematically evaluate the model’s recall performance against stored patterns, pattern overlap, network size, and active cells per pattern, we selectively modulated feedforward and feedback excitatory and inhibitory pathways targeting specific excitatory and inhibitory cells. Of the different model variations (modulated pathways) tested, ‘model 1’ recall quality was excellent across all conditions. ‘Model 2’ recall was the worst. The number of ‘active cells’ representing a memory pattern was the determining factor in improving the model’s recall performance regardless of the number of stored patterns and overlap between them. As ‘active cells per pattern’ decreased, the model’s memory capacity increased, interference effects between stored patterns decreased, and recall quality improved.

## Introduction

The case of Henry Molaison (the famous ‘HM’ patient) has taught us a great deal of what memory is and what is the cost of losing it [1]. At a very early age, HM experienced intractable epilepsy suffering from minor seizures, which became major in puberty. Later, in life and despite high doses of anticonvulsant medication, his seizures dominated his life, so no longer could work or lead a normal life. In an attempt to cure his seizures, clinicians recommended bilateral medial temporal lobectomy to surgically resect his hippocampi, parahippocampal cortices, entorhinal cortices, piriform cortices, and amygdalae [2]. Although the surgery was a success and HM was able to control his epilepsy, a severe side effect from the surgery gave him a profound anterograde amnesia, an inability to form new episodic or factual long-term memories, although his memories up-to-surgery remained intact. Thanks to HM and other patients [3], we now know how important memory is in our lives and what is the cost of losing it. Without memory, we cannot remember even our most basic experiences, such as what we had for breakfast or where did we park our car, let alone think about the future. Without memory, we cannot learn anything new.

HM has also taught us that the hippocampus is an important brain structure responsible for short-term storage of declarative memories [4]. It is a well-studied brain area from which a wealth of knowledge of cell types and their anatomical, physiological, synaptic, and network properties has been gained [5]. Its principal excitatory neurons are the pyramidal cells (PCs) in regions CA3 and CA1 and granule cells in dentate gyrus (DG). In addition to excitatory cells, hippocampus has a large variety of inhibitory interneurons [6–8]. Excitatory and inhibitory cells in the hippocampus form intricate microcircuits, which compute information differently in each hippocampal region. DG microcircuits have been implicated in pattern separation [9–12], whereas CA3 ones in pattern completion [9, 10] and CA1 ones in novelty detection [13] and mismatch of expectations [14]. These microcircuits exhibit also different rhythms which correlate positively with different behavioral conditions. Theta (4–7 Hz) and gamma (30–100 Hz) oscillations have been shown to co-exist [15] and their co-existence has been hypothesized to support specific functional information processing [16]. Theta oscillations have been implicated in episodic and spatial memory formation [17–22] and disruption of them results in behavioral deficits [23].

**Fig. 1:**
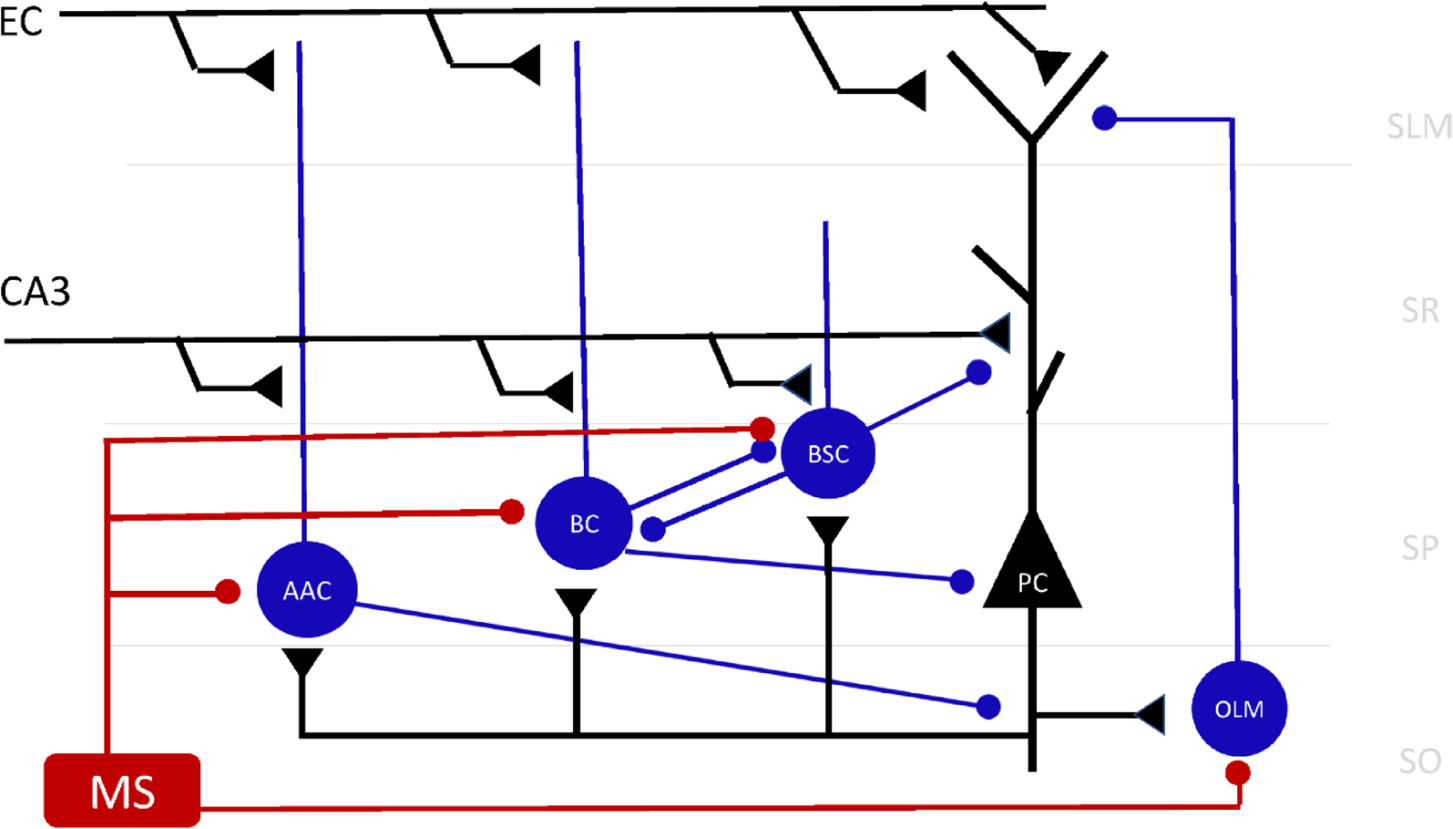
Hippocampal CA1 microcircuit showing major cell types and their connectivity. SLM: Stratum lacunosum-moleculare; SR: stratum radiatum; SP Stratum pyramidale; SO: stratum oriens; PC: pyramidal cell; AAC: axo-axonic cell; BC: basket cell; BSC: bistratified cell; CA3: CA3 Schaffer collateral input; MS: medial septum. Black lines: excitatory input; blue lines: inhibitory input; maroon lines: MS inhibitory input

In 2010, a detailed computational model of the CA1 microcircuit (Fig. 1) was first introduced which showed how memory formation (storage and recall) could be controlled [17]. The model was based on the many details we knew then of the neuronal hippocampal circuit. It showed how theta modulated inhibition separated encoding and retrieval of memories in CA1 into two functionally independent processes and predicted functional roles of various guises (somatic, axonic, and dendritic) of inhibition in these processes. In the model, somatic inhibition allowed generation of dendritic calcium spikes that promoted synaptic long-term potentiation (LTP), while minimizing cell output. Proximal dendritic inhibition controlled cell firing, prevented LTP by suppressing dendritic calcium spikes, and removed interference from spurious memories during recall, whereas distal dendritic inhibition removed interference from new memories been encoded during recall of old memories. The model’s memory capacity and recall performance was tested as more and more memories were stored in its synapses. Results showed that mean recall quality decreased as more memory patterns were loaded into the model’s synapses due to interference with previously stored ones.

In this article, we systematically investigated the biophysical mechanisms that could improve the memory capacity and recall performance of the Cutsuridis et al.’s model [17] by selectively modulating feedforward and feedback excitatory and inhibitory pathways targeting specific excitatory and inhibitory cells in the network model. Our work investigated ways to remove spurious activity and improve the mean recall quality of the network as a function of network size, stored patterns, pattern overlap, and number of active cells.

## Materials and methods

The complete computational CA1 microcircuit model of Cutsuridis et al. [17] is depicted in Fig. 1. The complete model consisted of 100 PCs, one axo-axonic cell (AAC), two basket cells (BCs), one BSC, and one oriens lacunosum-moleculare (OLM) cell. An entorhinal cortical (EC) excitatory input excited the distal dendrites of PCs, AAC, and BC, whereas an excitatory Schaffer collateral CA3 input excited the proximal dendrites of PCs, AAC, BCs, and BSC. A medial septum (MS) inhibitory input inhibited all inhibitory cells in the network and caused them to fire at specific phases of a theta rhythm.

**Fig. 2:**
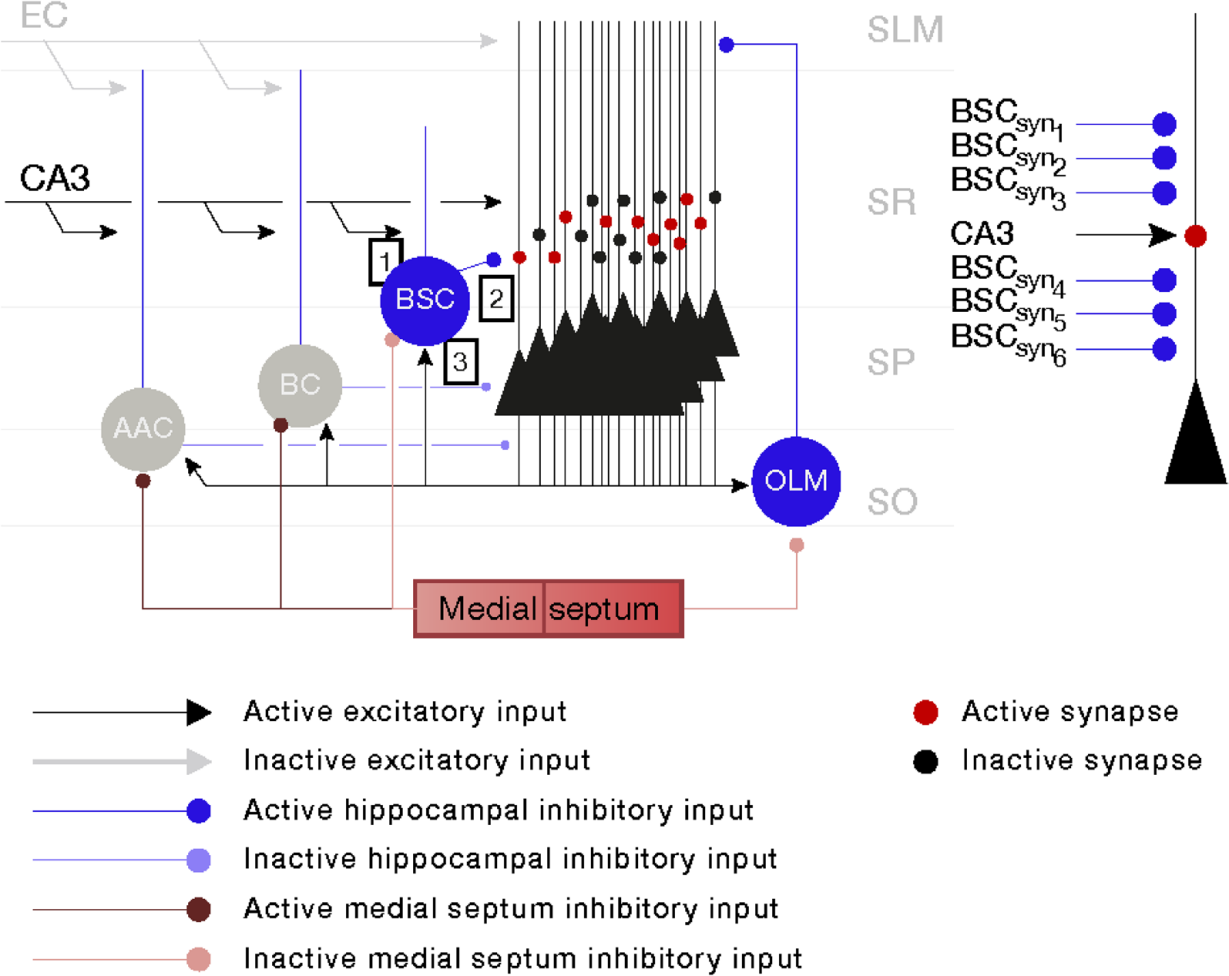
(Left) Recall microcircuit model of region CA1 of the hippocampus and (right) CA1-PC model with one excitatory (CA3) and six inhibitory (BSC) synaptic contacts on its dendrites. EC: entorhinal cortical input; CA3: Schaffer collateral input; AAC: axo-axonic cell; BC: basket cell; BSC: bistratified cell; OLM: oriens lacunosum-moleculare cell; SLM: stratum lacunosum-moleculare; SR: stratum radiatum; SP: stratum pyramidale; SO: stratum oriens. During recall, only PCs, BSC, and OLM cell are active. AAC and BCs are inactive due to strong medial septum inhibition. BSC and PC are driven on their SR dendrites by a strong CA3 excitatory input, which represented the contextual information. EC input is disconnected from the network, which thus has no effect on it. Red circles on PC dendrites represent loaded synapses, whereas black circles on PC dendrites represent unloaded synapses

**Fig. 3:**
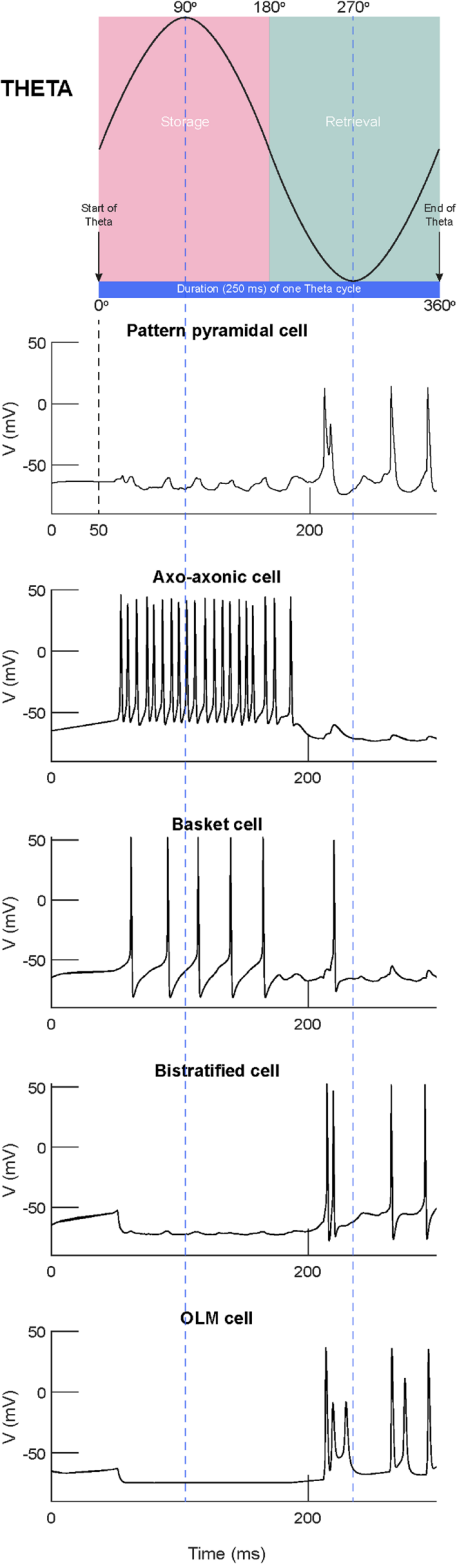
Voltage traces of model cells with respect to a single theta cycle

In this study where only the recall ability of the microcircuit was tested when a growing number of memory patterns were stored in its synapses without examining the exact details of the learning (storage) process, a sub-network of the complete microcircuit model was utilized. The sub-network consisted of *N* PCs (*N* = 100 or 300), one BSC, and one OLM cell (see Fig. 2). Simplified morphologies including the soma, apical and basal dendrites and a portion of the axon, were used for each cell type. The biophysical properties of each cell were adapted from cell types reported in the literature, which were extensively validated against experimental data in [24–27]. BCs and AAC although present in the network were disconnected from it and they were inactive during the retrieval cycle due to strong MS inhibition and hence had no effect on the network dynamics. EC input although present also had no effect on the network cells, because it was also disconnected. The only excitation to the network was from CA3 which excited the dendrites of BSC and PCs. All simulations were performed using NEURON [28] running on a PC with 4 CPUs under Windows 8. Voltage traces of all connected (PCs, BSC, OLM) and disconnected (AAC, BCs) cells to the network with respect to a single theta cycle are depicted in Fig. 3.

To assist the readers of this work and increase the readability of our manuscript, we provide below brief descriptions of each network’s components. Interested readers should refer to [17, 29] studies for more details of the microcircuit model and its components including the dimensions of their cells’ somatic, axonic, and dendritic compartments and distributions of passive and active conductances, synaptic waveforms, and synaptic conductances along these compartments. The complete mathematical formalism of the model can be found in the supplementary online materials document of [29].

### Pyramidal cells

Each PC had 15 compartments. Each compartment contained a calcium pump and buffering mechanisms, calcium activated slow after-hyperpolarized (AHP) and medium AHP K^+^ currents, a high-voltage activated (HVA) L-type Ca^2+^ current, an HVA R-type Ca^2+^ current, a low-voltage activated (LVA) T-type Ca^2+^ current, an h current, a fast sodium and a delayed rectifier K^+^ current, a slowly inactivating M-type K^+^ current, and a fast inactivating A-type K^+^ current [24, 25].

Each PC received mid-dendritic excitation from Schaffer collaterals (CA3-PCs), proximal excitation from around 1% of other CA1 PCs in the network (recurrent collaterals) [30], spatially distributed (six contacts) proximal dendritic synaptic inhibition from the BSC, and distal synaptic inhibition on each distal [stratum lacunosum-moleculare (SLM)] dendritic branch from the OLM cell.

### Bistratified cell

The BSC had 13 compartments. Each compartment contained a leak conductance, a sodium current, a fast delayed rectifier K^+^ current, an A-type K^+^ current, L- and N-type Ca^2+^ currents, a Ca^2+^-dependent K^+^ current, and a Ca^2+^- and voltage-dependent K^+^ current [26]. It received excitation from the CA3 Schaffer collaterals in its medial dendritic compartments, excitation from active CA1 PCs in its basal dendrites, and inhibition from MS in its basal dendritic compartments.

### OLM cell

The OLM cell had four compartments. Each compartment had a sodium (Na^+^) current, a delayed rectifier K^+^ current, an A-type K^+^ current, and an h current [27]. It received excitation from the PCs in its basal dendrites and inhibition from MS in the soma.

### Model inputs

**Fig. 4:**
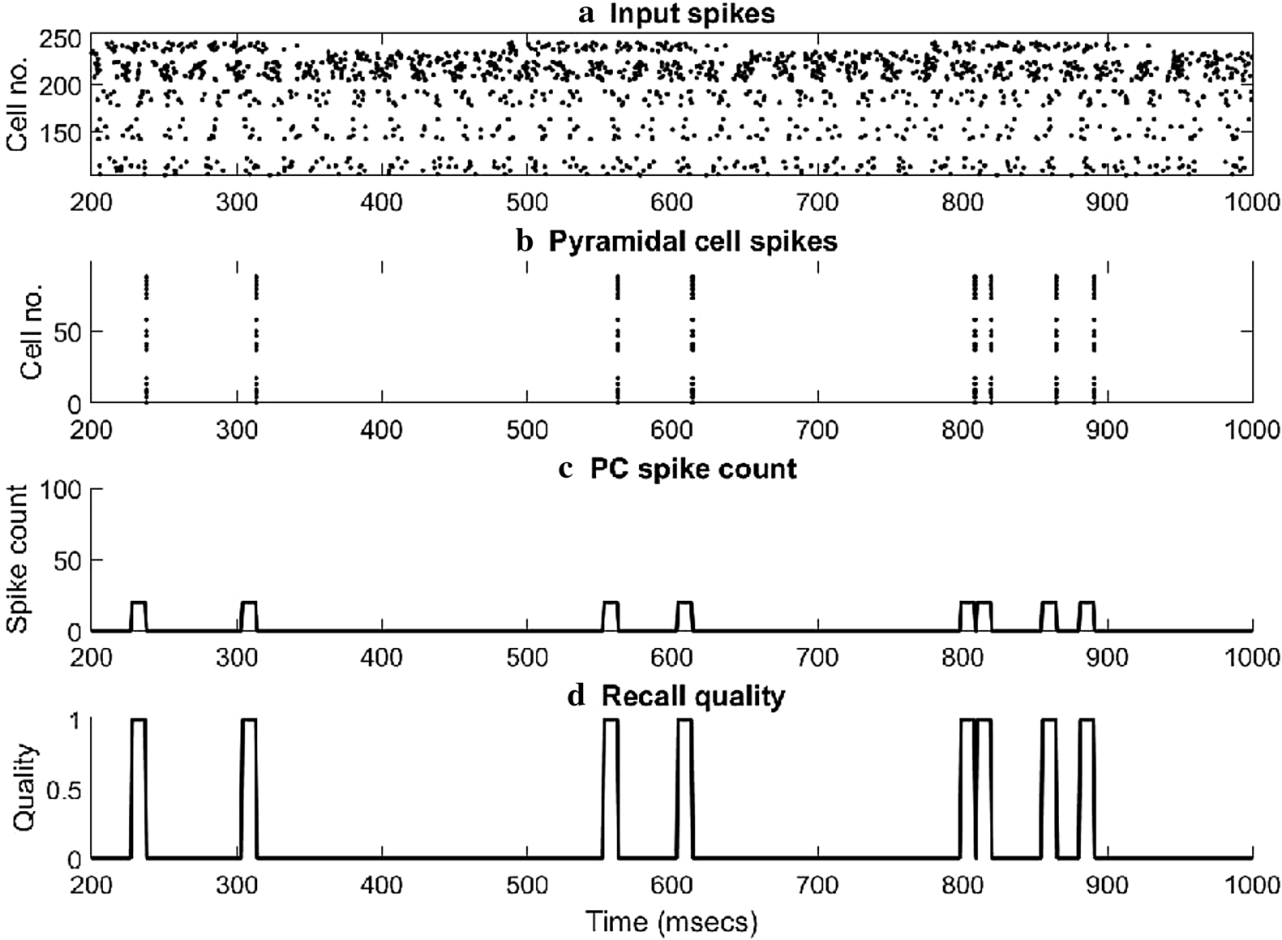
**a** Raster plot showing septal (top 20) and CA3 input (bottom 100) spikes. **b** Raster plot showing 20 ‘active cells’ activity coding for a particular memory pattern. **c** Twenty ‘active cells’ spike count in a sliding 10 ms bin. **d** Recall quality in a sliding 10 ms bin

An excitatory input originating from CA3 Schaffer collateral pyramidal cell axons and an inhibitory input originating from MS drove the network’s cells during recall (see Fig. 4). The CA3 input was modelled as the firing of *M* (*M* = 5, 10 or 20) out of *N* (*N* = 100 or 300) CA3 pyramidal cells at an average gamma frequency of 40 Hz (spike trains only modelled and not the explicit cells). PCs and BSC received CA3 excitatory input in their medial dendrites. MS inhibition was modelled as the rhythmic firing of two populations of ten septal cells (MS_1_ and MS_2_) each modulated at opposite phases of a theta cycle (180 ° out of phase) [31] (see Fig. 4). Each septal cell output was modelled as bursts of action potentials using a presynaptic spike generator. Each spike train consisted of bursts of action potentials at a mean frequency of 8 Hz for a half-theta cycle (70 ms) followed by a half-theta cycle of silence. Due to 8% noise in the inter-spike intervals, the ten spike trains in each septal population were asynchronous. During recall, MS_1_ cells inhibited the MS_2_ cells, which dis-inhibited the BSC and OLM cells in the network.

### Synaptic properties

AMPA, NMDA, GABA-A, and GABA-B synapses were considered. GABA-A were present in somatic and dendritic compartments, whereas GABA-B were present only in medial and distal dendrites. AMPA and NMDA synapses were present only in medial dendrites.

### Network testing

The goal of this research work was to test the recall performance of the model when the network had already stored patterns without examining the exact details of the learning process. To test the recall performance of the model, the methodology described in [17] was adopted. A memory pattern was stored by generating a weight matrix based on a clipped Hebbian learning rule. This weight matrix was used to prespecify the CA3–CA1 PC connection weights. Without loss of generality, the input (CA3) and output (CA1) patterns were assumed to be the same, with each pattern consisting of *M* (*M* = 5, 10 or 20) randomly chosen PCs (active cells) out of the population of *N* (*N* = 100 or 300) PCs. The *N* × *N* (*N* × *N* = 100 × 100 or 300 × 300)-dimensional weight matrix was created by setting matrix entry (*i*, *j*), *w*_*ij*_ = 1 if input PC *i* and output PC *j* are both active in the same pattern pair; otherwise, weights are 0. Any number of pattern pairs could be stored to create this binary weight matrix. The matrix was applied to our network model by connecting a CA3 input to a CA1 PC with a high AMPA conductance (gAMPA = 1.5 nS) if their connection weight was 1, or with a low conductance (gAMPA = 0.5 nS) if their connection was 0. This approach is supported by experimental evidence favouring two-state synapses [32].

### Memory patterns

**Fig. 5:**
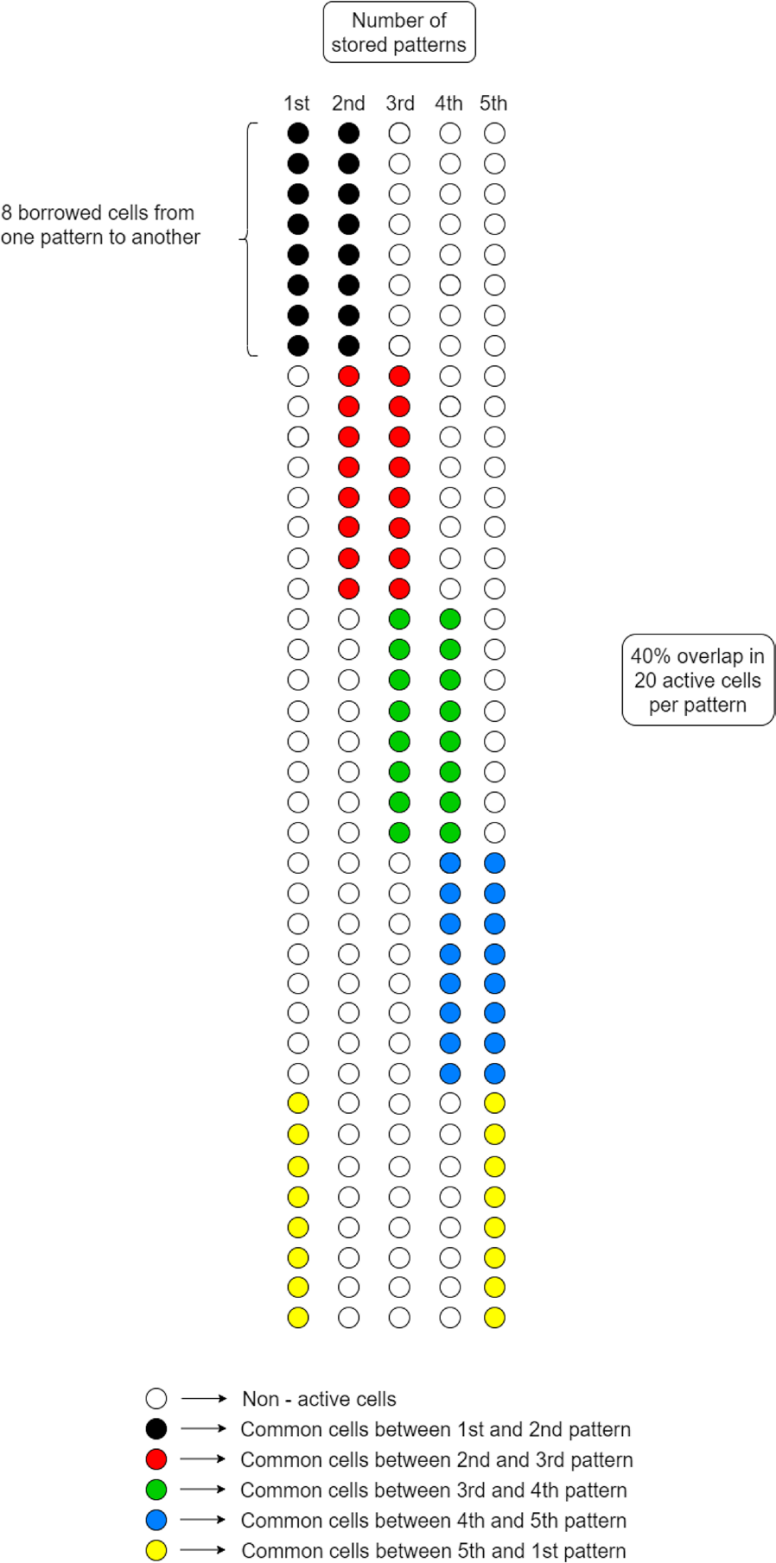
Exemplar set of five memory patterns with 40% overlap between them

Sets of memory patterns at different sizes (1, 5, 10, 20), pattern overlaps (0%, 10%, 20%, 40%), and number of active cells per pattern (5, 10, 20) were created. A 0% overlap between for example five patterns in a set meant no overlap between patterns 1 and 2, 1 and 3, 1 and 4, 1 and 5, 2 and 3, 2 and 4, 2 and 5, 3 and 4, 3 and 5, and 4 and 5. Similarly, a 40% overlap between five patterns in a set meant that 0.4 × *Μ* cells were shared between patterns 1 and 2, a different 0.4 × *Μ* cells were shared between patterns 2 and 3, a different 0.4 × *Μ* cells between patterns 3 and 4, a different 0.4 × *Μ* cells between patterns 4 and 5, and a different 0.4 × *Μ* cells between patterns 5 and 1 (see Fig. 5). For 20 active cells per pattern, a maximum of five patterns could be stored by a network of 100 PCs. For ten active cells per pattern, a maximum of ten patterns could be stored, and for five active cells per pattern, a maximum of 20 patterns could be stored. Similar maximum number of patterns could be stored for 10%, 20%, and 40% overlap and 5, 10, and 20 active cells per pattern, respectively.

### Recall performance

To measure the recall performance of our network, the normalized dot product metric was used which measured the distance between the recalled output pattern, *A*, from the required output pattern, A^*^:

1 $$C=\frac{A\cdot {A}^{*}}{\left({\sum }_{i=1}^{{N}_{A}}{A}_{i}\cdot {\sum }_{j=1}^{{N}_{A}}{A}_{j}^{*}\right)},$$

where *N*_*A*_ is the number of output units. Correlation between the recalled and required output patterns took value from 0 (no correlation between output pattern *A* = [1 0 1 0 1 0] and output pattern A^*^ = [0 1 0 1 0 1]) to 1 (output pattern *A* = [1 0 1 0 1 0] and output pattern A^*^ = [1 0 1 0 1 0] are identical). The higher the correlation value, the better the recall performance.

### Mean recall quality

We defined mean recall quality of our network model as the mean value of all recall qualities estimated from each pattern presentation when a *P* number of patterns were already stored in the network:

2 $$\mathrm{MC}=\frac{\sum_{i=1}^{{N}_{p}}{C}_{i}}{{N}_{p}},$$

where *C*_*i*_ is the recall quality of pattern *i* and *N*_*p*_ is total number of recalled patterns. For example, when ten patterns (*N*_*p*_ = 10) were initially stored in the network and pattern 1 was presented to the network during recall, then a recall quality value for pattern 1 (C1) was calculated. Repeating this process for each of the other patterns [pattern 2 (C2), pattern 3 (C3), …, pattern 10 (C10)], a recall quality value was calculated. The mean recall quality (MC) of the network was then the mean value of these individual recall qualities.

### Model selection

In [17], BSC inhibition to PC dendrites acted as a global non-specific threshold machine capable of removing spurious activities at the network level during recall. BSC inhibition was held constant as more patterns loaded onto the PC dendritic synapses. The recall quality of the model in [17] decreased as more and more memories were stored (see Fig. 14 in [17]).

**Fig. 6:**
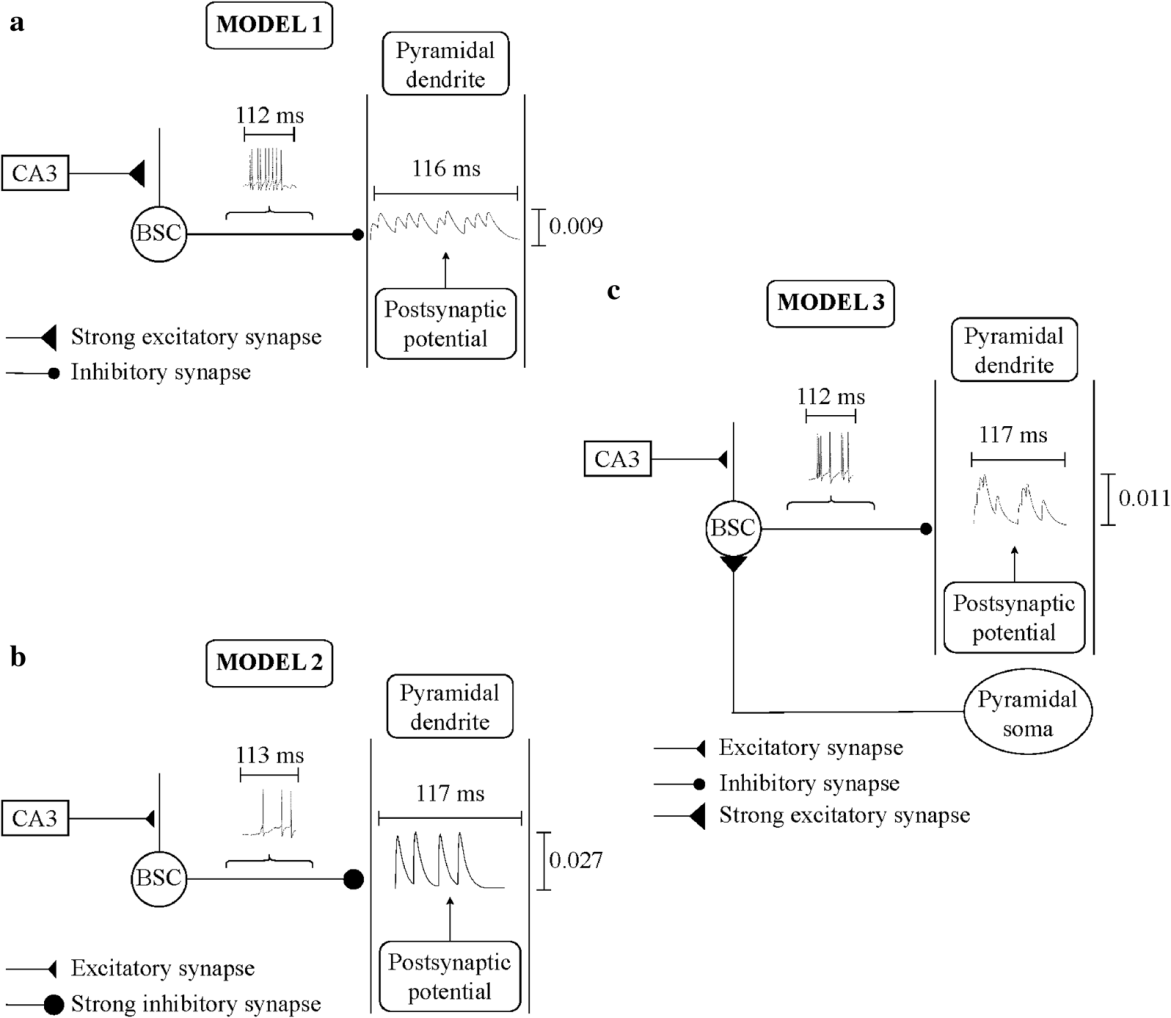
Schematic drawing of **a** ‘model 1’, **b** ‘model 2’, and **c** ‘model 3’. In ‘model 1’, a strong excitatory CA3 input increases BSC firing response, which generates on PC dendrite numerous small amplitude IPSPs, thus producing a very strong inhibitory environment which filters out spurious neuronal activities. In ‘model 2’ a strong BSC inhibitory drive to PC dendrite causes postsynaptically fewer, but with larger amplitude IPSPs. In ‘model 3’, a strong excitatory PC feedback signal to BSC increases its firing response, which generates fewer than ‘model 1’ IPSPs on PC dendrite, and hence a less strong inhibitory environment than ‘model 1’

To improve the recall performance of [17], we artificially modulated the synaptic strength of selective excitatory and inhibitory pathways to BSC and PC dendrites as more and more patterns were stored in the network:

1. Model 1: strengthening of CA3 feedforward excitatory synaptic drive to BSC dendrites (Fig. 6a) increased BSC’s firing rate. As a result, more IPSPs were generated in the PC dendrites producing a very strong inhibitory environment, which eliminated all spurious activity.
2. Model 2: strengthening of BSC feedforward inhibitory synaptic drive to PC dendrites (Fig. 6b) produced fewer IPSPs, but with greater amplitude.
3. Model 3: strengthening of PC feedback excitatory synaptic drive to BSC basal dendrites (Fig. 6c) had a similar effect as model 1, but with smaller potency.

## Results

A set of patterns (1, 5, 10, 20) at various percent overlaps (0%, 10%, 20%, 40%) were stored by different number of ‘active cells per pattern’ (5, 10, 20) without recourse to a learning rule by generating a weight matrix based on a clipped Hebbian learning rule, and using the weight matrix to prespecify the CA3 to CA1 PC connection weights. To test recall of a previously stored memory pattern in the model, the entire associated input pattern was applied as a cue in the form of spiking of active CA3 inputs (those belonging to the pattern) distributed within a gamma frequency time window. The cue pattern was repeated at gamma frequency (40 Hz). During the retrieval, only the BSCs and OLM cells were switched on, whereas the AACs and BCs were switched off. The CA3 spiking drove the CA1 PCs plus the BSCs. The EC input, which excited the apical dendrites of PCs, AACs, and BCs, was disconnected during the retrieval.

### Recall quality as a function of pattern overlap

**Fig. 7:**
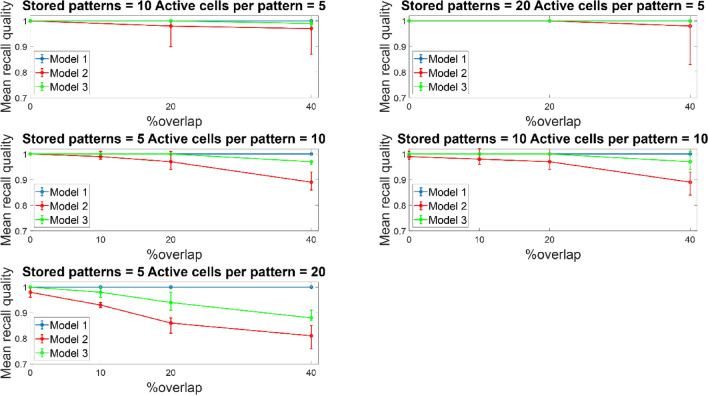
Mean recall quality of ‘model 1’, ‘model 2’, and ‘model 3’ as a function of percent overlap (0%, 10%, 20%, 40%). Each model was a network of 100 PCs, 1 BSC and 1 OLM cell

Figure 7 depicts the mean recall quality of all three tested models (‘model 1’, ‘model 2’, ‘model 3’) as a function of percent overlap between stored patterns. Recall quality was best for all three models (‘model 1’, model 2’, ‘model 3’) when overlap was small (up to 10%) regardless of the number of ‘active cells per pattern’ (i.e., the number of cells needed to represent a memory pattern) and patterns stored in the network. For pattern overlaps greater than 10%, recall quality depended solely on the number of ‘active cells’ representing a pattern and it was independent of how many patterns were stored in the network. When five ‘active cells’ were used to represent a memory, then recall quality was best for all three models across all overlaps and irrespective of memory patterns stored. When ten ‘active cells’ were used to represent a memory, the performances of all three models were comparably similar when five or ten patterns were stored and across overlap percentages. When 20 ‘active cells’ were used to represent a memory, then even for just five patterns stored, the recall quality for ‘model 2’ was consistently worst across all overlaps. The performance of ‘model 1’ was consistently best across all condition, whereas the performance of ‘model 3’ was between ‘model 1 and 2’. The performances of ‘model 2’ and ‘model 3’ get worse as overlap increased (from 10 to 40%).

**Fig. 8:**
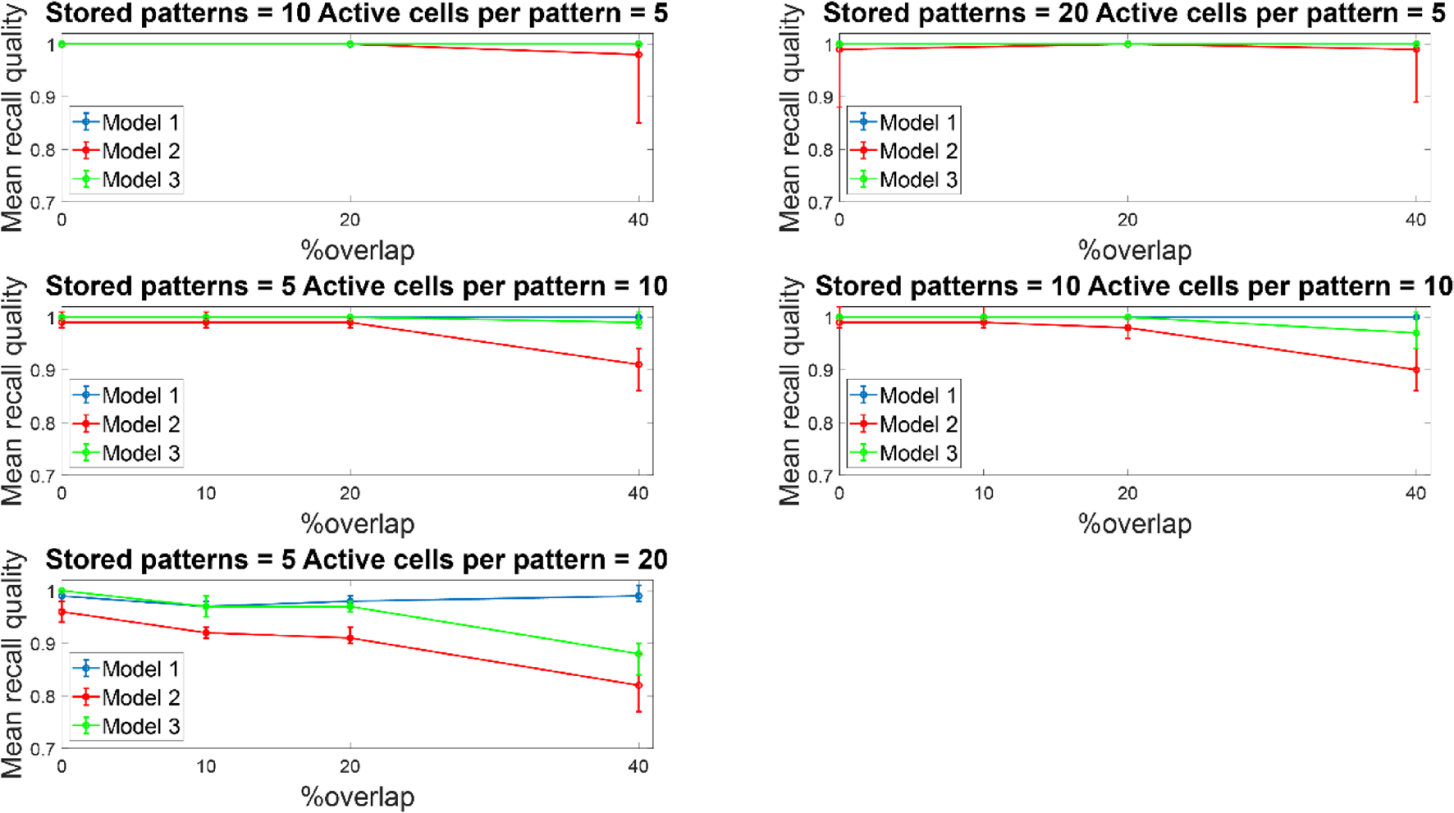
Mean recall quality of ‘model 1’, ‘model 2’, and ‘model 3’ as a function of percent overlap (0%, 10%, 20%, 40%). Each model was a network of 300 PCs, 1 BSC, and 1 OLM cell

**Fig. 9:**
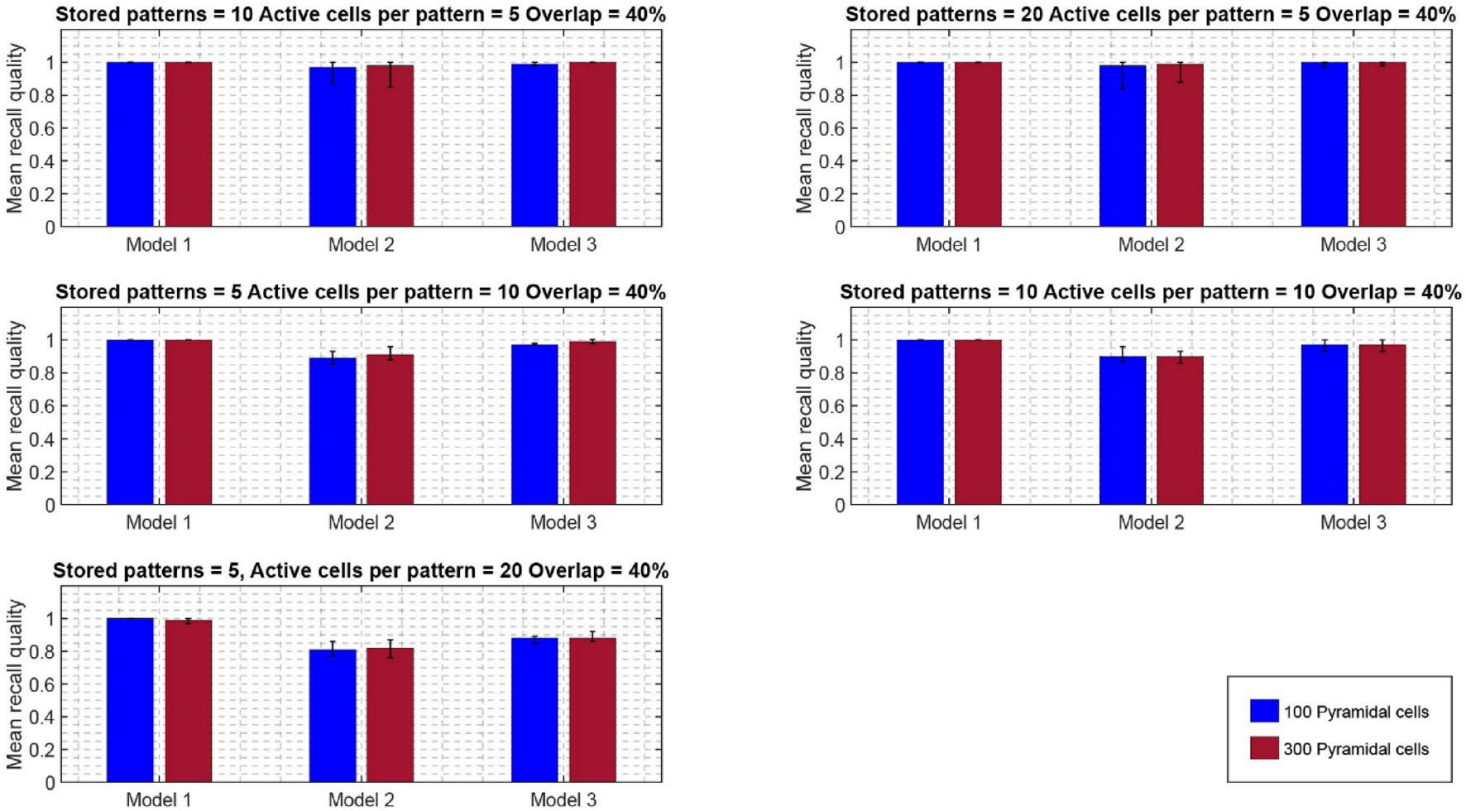
Comparison of mean recall quality of ‘model 1’, ‘model 2’, and ‘model 3’ as a function of network size (100 PCs vs 300 PCs) for different numbers of stored patterns, active cells, and 40% pattern overlap

Similar recall performances were observed when the network size increased from 100 to 300 PCs, while keeping all other network components the same (one BSC, six inhibitory synaptic contacts of BSC onto PCs dendrites, one OLM cell) (see Fig. 8). When the network size increased, a performance improvement was evident even at 20% overlap for all three models and across conditions (active cells per pattern, and stored patterns). At larger overlaps as with the smaller network, the determinant factors for excellence in performance were the number of ‘active cells per pattern’, and modulated pathway. ‘Model 1’ (i.e., strengthening of CA3 feedforward excitatory synaptic drive to BSC dendrites) was consistently best across all conditions (active cells per pattern, stored patterns, % overlaps) and against other models (‘model 2’ and ‘model 3’). A direct comparison of the mean recall quality of the small network (100 PCs) against the large network (300 PCs) showed no significant differences across all conditions (see Fig. 9).

### Recall quality as a function of active cells

**Fig. 10:**
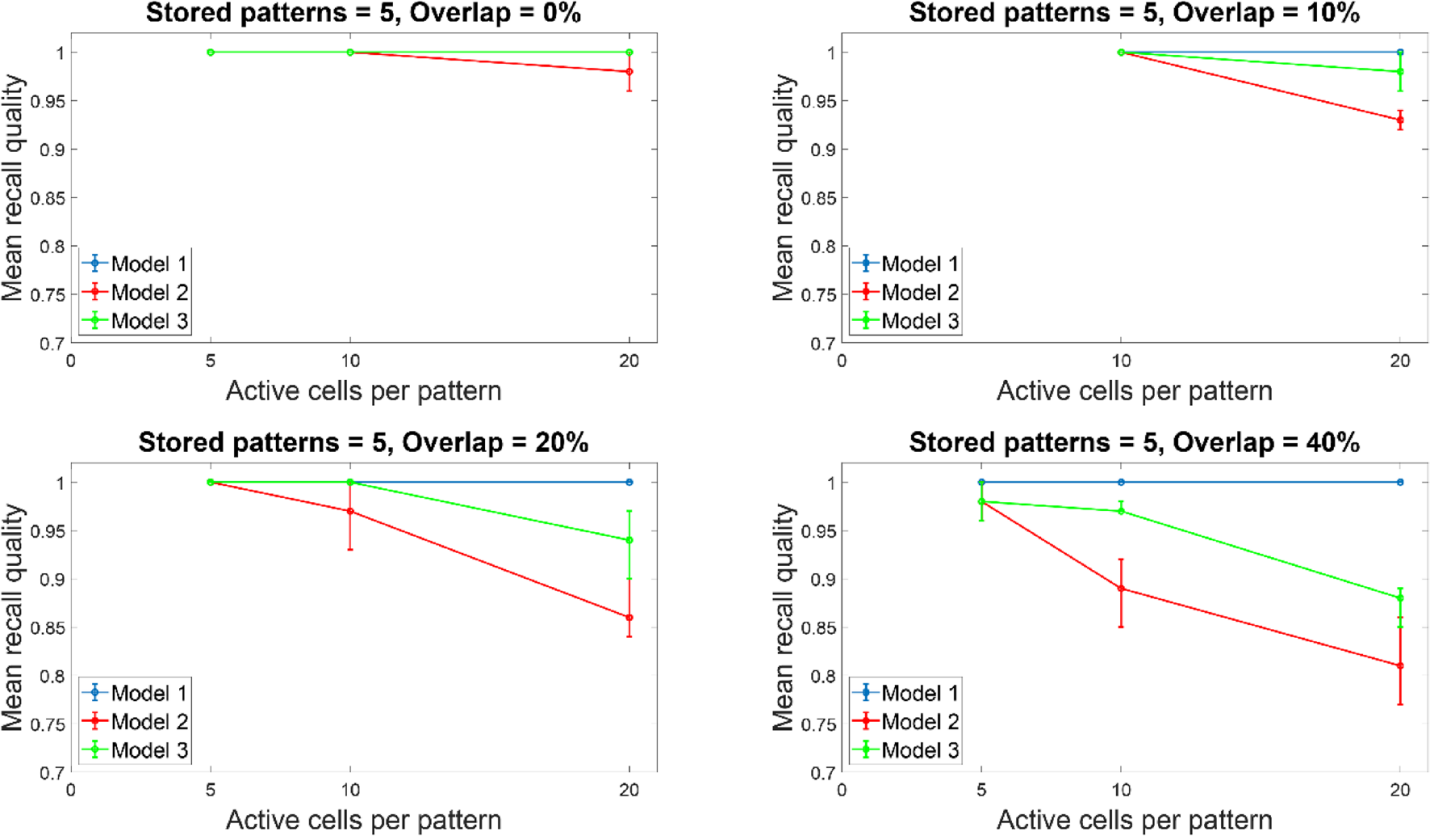
Mean recall quality of ‘model 1’, ‘model 2’, and ‘model 3’ as a function of active cells per pattern (5, 10, 20) when five patterns were stored with various percentages of pattern overlap. Each model was a network of 100PCs, 1 BSC, and 1 OLM cell

**Fig. 11:**
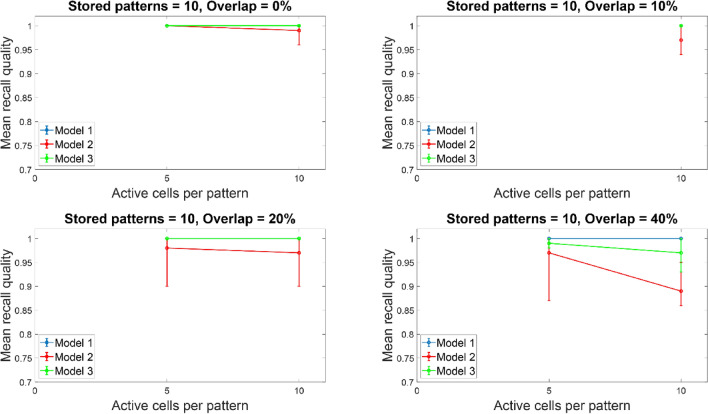
Mean recall quality of ‘model 1’, ‘model 2’, and ‘model 3’ as a function of active cells per pattern (5, 10, 20) when ten patterns were stored with various percentages of pattern overlap. Each model was a network of 100PCs, one BSC,, and one OLM cell

Figures 10 and 11 depict the mean recall quality of models 1–3 of the small network (100 PCs) against number of ‘active cells per pattern’ for various overlaps (0%, 10%, 20%, and 40%) when five (Fig. 10) and ten (Fig. 11) patterns were stored in the network. When five or ten ‘active cells’ were used to represent a memory, then the recall performances of all three models when number of stored patterns were five or ten were comparable at 0%, 10%, 20%, and 40%, respectively. This meant that the number of patterns stored in the network did not had any effect in its recall quality. When ‘active cells’ were increased (from 10 to 20), then the recall qualities of models 2 and 3 progressively got worse as overlap between patterns increased (from 0 to 40%). ‘Model 1’ recall quality was consistently best (*C* = 1) across ‘active cells’, stored patterns, and overlap conditions.

**Fig. 12:**
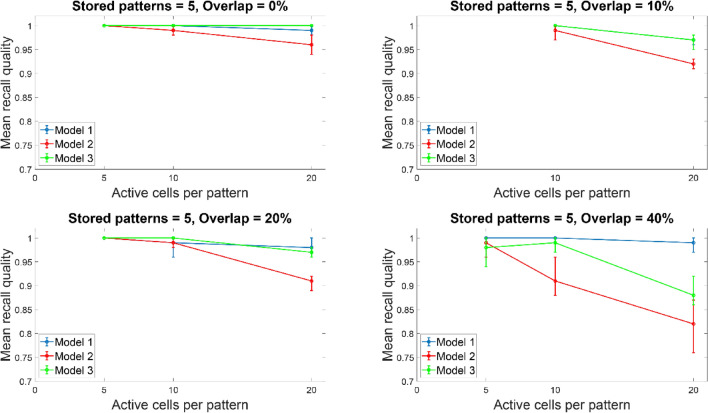
Mean recall quality of ‘model 1’, ‘model 2’, and ‘model 3’ as a function of active cells per pattern (5, 10, 20) when five patterns were stored with various percentages of pattern overlap. Each model was a network of 300PCs, one BSC, and one OLM cell

**Fig. 13:**
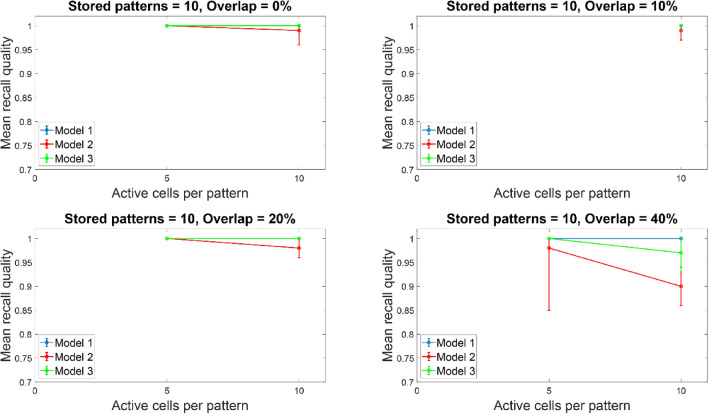
Mean recall quality of ‘model 1’, ‘model 2’, and ‘model 3’ as a function of active cells per pattern (5, 10, 20) when 10 patterns were stored with various percentages of pattern overlap. Each model was a network of 300 PCs, one BSC, and one OLM cell

Figures 12 and 13 depict the mean recall qualities of models 1–3 of the large network (300 PCs) against number of ‘active cells per pattern’ for various overlaps (0%, 10%, 20%, and 40%) when five (Fig. 12) and ten (Fig. 13) patterns were stored in the network. Comparable recall quality results to the smaller network are evident. As before the number of stored patterns had a minor effect on the recall quality of the network (‘model 1’, ‘model 2’, and ‘model 3’). When ‘active cells per pattern’ were increased (from 10 to 20), then the recall qualities of models 2 and 3 progressively got worse as overlap between patterns increased (from 0 to 40%). As before, ‘model 1’ recall quality was consistently best (*C* = 1) across ‘active cells per pattern’, stored patterns, and overlap conditions.

**Fig. 14:**
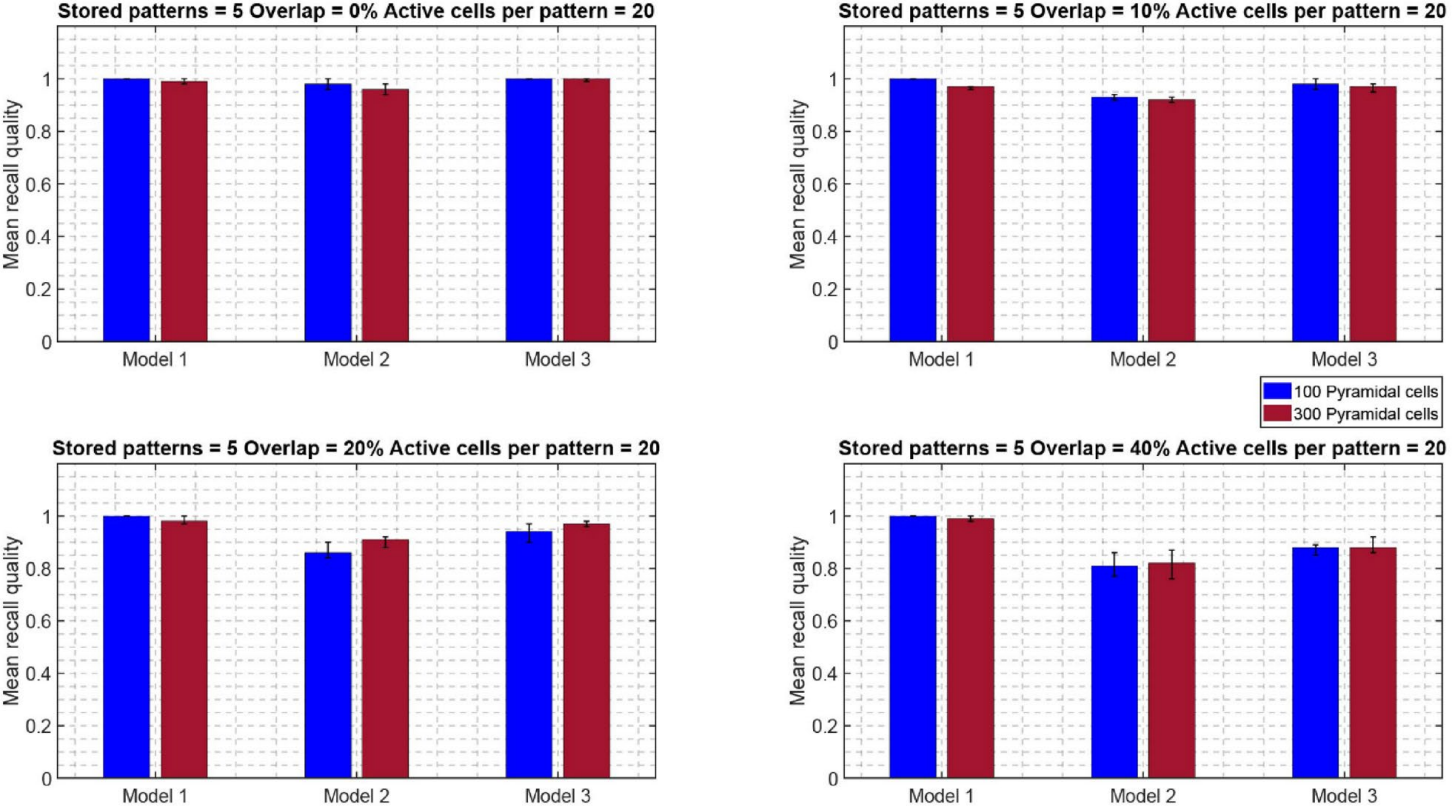
Comparison of mean recall quality of ‘model 1’, ‘model 2’, and ‘model 3’ as a function of network size (100 PCs vs 300 PCs) for five stored patterns, 20 active cells, and different % pattern overlaps

**Fig. 15:**
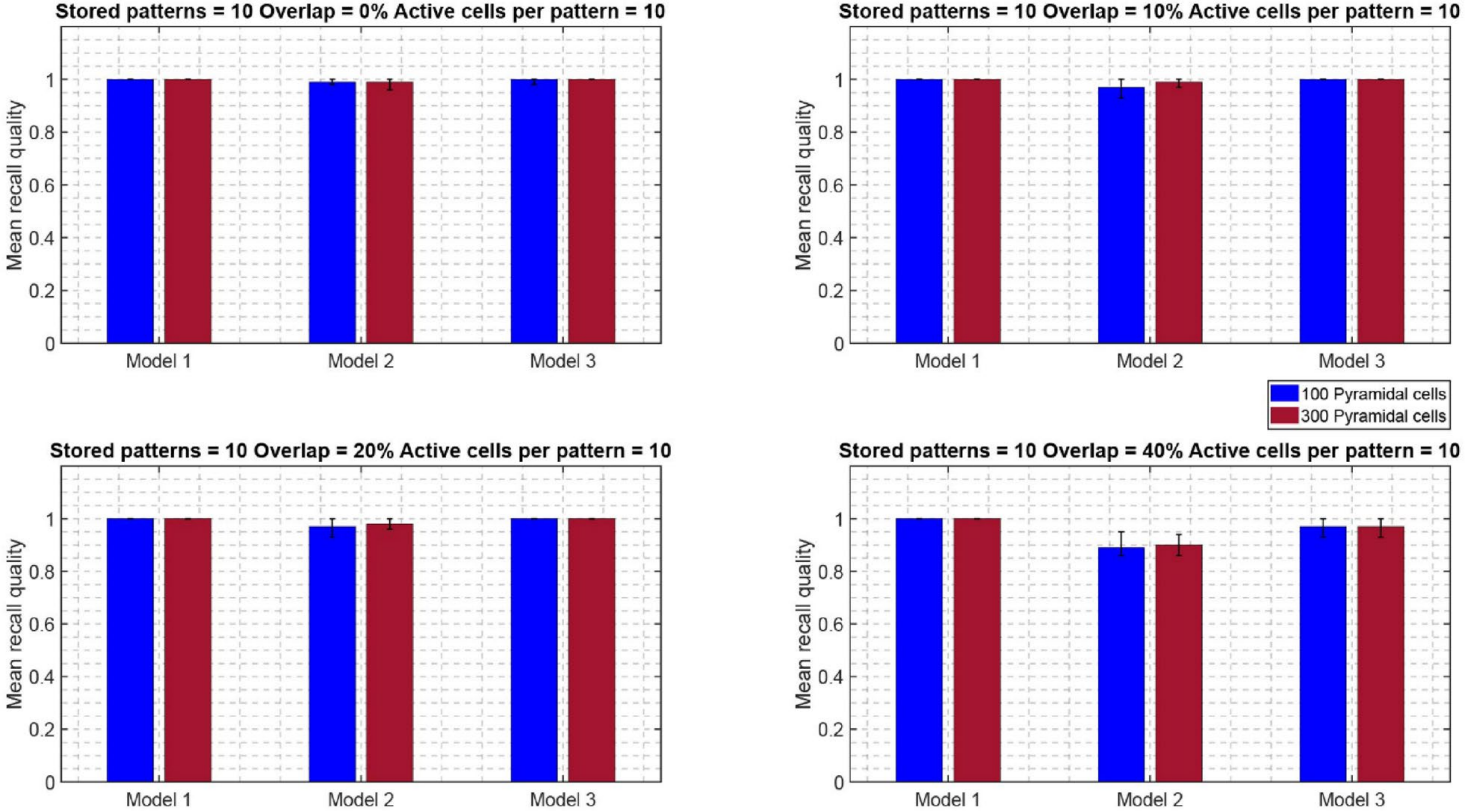
Comparison of mean recall quality of ‘model 1’, ‘model 2’, and ‘model 3’ as a function of network size (100 PCs vs 300 PCs) for ten stored patterns, ten active cells, and different % pattern overlaps

Direct comparisons of the mean recall quality of the smaller network (100 PCs) against the larger network (300 PCs) when five (Fig. 14) and ten (Fig. 15) patterns were stored showed no significant differences across all conditions.

## Discussion

### General model considerations

A biologically realistic microcircuit model of region CA1 of the hippocampus with morphologically simplified neurons was employed [17, 29]. The model [17] demonstrated that encoding and retrieval of memories can be separated into two independent theta half-cycles paced by theta modulated intra- and extra-hippocampal inhibition. The model simulated the timing of different extra- and intra-hippocampal cells types relative to the theta rhythm in anesthetized animals [31, 33, 34]. Out of the possible excitatory and inhibitory pathways affecting the network’s dynamics, we selected in this study to quantitatively modulate the following three pathways: (1) strengthening of the feedforward CA3 excitatory synaptic drive to BSC dendrites (‘model 1’), (2) strengthening of the feedforward BSC inhibitory synaptic drive to PC dendrites (‘model 2’), and (3) strengthening of the feedback PC excitatory drive to BSC basal dendrites (‘model 3’).

### Outstanding questions of memory research our model addressed

A number of outstanding questions of memory research were addressed by our study. What constitutes a memory at the network level? How many ‘active cells’ can accurately represent a memory pattern and how do these ‘active cells’ affect the recall performance of the network? Is the network’s performance affected as more and more memory patterns with greater degrees of overlap are stored in the network? How does an increase in network size affect the network’s recall performance?

Our study showed that a memory pattern at the network level can be represented by a high gamma coordinated activity of a population of cells (called here ‘active cells per pattern’) (see Fig. 4b). We found that the number of ‘active cells’ coding for a memory pattern was a key determinant factor for improving the recall performance of each model tested. Our simulations showed that as the number of ‘active cells’ coding for a memory pattern decreased, then the better the recall performance of the model was regardless of how many patterns were stored and the degree of overlap between them. This was the case, because as fewer active cells represented a memory, even though many patterns were stored in the network, then the network’s memory weight matrix was dominated with fewer ‘1 s’ and more ‘0 s’, and hence, interference between the stored patterns was not so strong to decrease the network’s recall performance. As soon as the number of ‘active cells’ coding for a memory pattern was increased, then the network weight matrix was populated with more ‘1 s’ and fewer ‘0 s’, and hence, interference effects between stored patterns took over and the network’s recall performance decreased. We found the latter finding to be also depended on the chosen model. ‘Model 1’ performance was excellent across all conditions (‘active cells’, ‘stored patterns’, ‘degree of overlap’, ‘network size’), whereas ‘model 2’ performance was the worst and ‘model 3’ performance was somewhere between ‘model 1 and 2’.

Our simulations further showed that the effect of the degree of overlap between stored memory patterns was heavily depended on the modulated pathway (i.e., the model tested). As degree of overlap increased (from 10 to 40%), the performances of ‘model 2’ and ‘model 3’ got worse. This finding was independent of the number of patterns stored in the network. An increase in the network size (from 100 to 300 PCs) had ‘no effect’ on average on the recall performances of the three models tested. This was because although we increased the number of PCs in the network, we kept all other network components (one BSC, one OLM, six BSC inhibitory synaptic contacts onto the PC dendrites) and conditions (‘active cells’, ‘stored patterns’, ‘pattern overlap’) the same. As we mentioned above, in the smaller network (100PCs), its weight matrix was not saturated (i.e., it had fewer ‘1 s’ and more ‘0 s’), so an increase in network size and in network’s weight matrix from 100 × 100 to 300 × 300 dimensions had a very little effect on the network’s performance.

So why was ‘model 1’ performance so consistently better than ‘model 2’ and ‘model 3’ across all conditions? Why the recall performance of ‘model 1’ was always so outstanding even when more and more patterns were stored, less or more ‘active cells per pattern’ were utilized and greater degree of overlap between patterns was used? As we described in Sect. 2.10, ‘model 1’ was the case where CA3 feedforward excitatory drive to BSC was strengthened, causing the BSC’s firing rate to increase. As a result, more IPSPs were generated in the PC dendrites producing a very strong inhibitory environment (‘cloud’), which eliminated all spurious activity (see Fig. 6a). ‘Model 3’ was the case the PC feedback excitatory drive to BSC basal dendrites increased as more and more patterns were stored, causing the BSC’s firing rate to also increase but not as much as in ‘model 1’. As a result, IPSPs were generated in the PC dendrites producing though a less strong inhibitory cloud, which eliminated most spurious activity, but not all (see Fig. 6c). ‘Model 2’ was the case where strengthening of BSC feedforward inhibitory synaptic drive to PC dendrites produced fewer IPSPs on them, but with greater amplitude (see Fig. 6b). In all simulations, ‘model 1’ outperformed ‘model 3’ across all conditions (overlaps and ‘active cells per pattern’). The main reason for such an outstanding performance of ‘model 1’ was because the BSC dendrites were excited by a higher frequency (40 Hz) excitatory drive (100 CA3-PCs), whereas in ‘model 3’, BSC dendrites were excited by a much lower frequency excitatory drive originating from the *M* ‘active cells’ (CA1-PCs) that represented the pattern (CA1 PCs fired once or twice per retrieval cycle). Since in ‘model 1’, the BSC firing frequency response was higher than in ‘model 3’, then the postsynaptic effect the BSC had on the PC dendrites in ‘model 1’ was higher in frequency and duration (but not in amplitude) than in ‘model 3’ (see Fig. 6a, c). Thus, ‘model 1’ had a better success at removing spurious activities and improving recall quality than ‘model 3’. Since the BSC frequency response in ‘model 2’ was fixed, but its postsynaptic effect (weight) on PC dendrites was strengthened, then the amplitude of the inhibitory postsynaptic potentials (IPSPs) on PC dendrites increased (compared to the IPSP amplitudes in models 1, 3), but their frequency responses were low (lower than in models 1, 3; see Fig. 6b). Each IPSP decayed to almost zero before another IPSP was generated postsynaptically on the PC dendrites.

### Future extensions

Several extensions to the basic idea deserve further consideration. Although in this study, only three excitatory and inhibitory pathways were selected to be modulated to examine their effects on the recall performance of the CA1 microcircuit model, there are several other pathways that can be modulated (e.g., a combination of ‘model 1’ with ‘model 3’, or a combination of ‘model 2’ with ‘model 3’, etc.). Another idea worth pursuing further is to examine quantitatively the effect of the BSC inhibitory cloud on the PC dendrites. What will be the effect on the network’s recall performance if the potency of the postsynaptic inhibitory cloud is decreased or increased? What will be its effect when OLM inhibition is removed from the network? What will be the network’s recall performance when the same memory pattern that is retrieved is also being encoded? What will the effect when a new memory pattern is trying to be encoded when an old one is being retrieved? Under what network conditions (network size, active cells, stored patterns, etc.) will the performance of ‘model 2’ become better? These are some of the questions our research is currently attempting to address.

## Conclusions

A bio-inspired microcircuit model of region CA1 region of the hippocampus [5] was employed to systematically evaluate its mean recall quality against growing numbers of stored patterns, increased percentages of overlaps, and decreasing numbers of ‘active cells per pattern’. The strengths of three selective excitatory and inhibitory pathways to BSC and PC dendrites were chosen to be strengthened as more and more patterns were stored in the network, which resulted in three different network models, the performances of which were compared against each other. The recall performance of ‘model 1’ was found to be excellent (*C* = 1) across all conditions, whereas the recall performance of ‘model 2’ was the worst. One of our key findings of our study was that the number of ‘active cells per pattern’ had a massive effect on the recall quality of the network regardless of how many patterns were stored in it. As the number of dedicated cells representing a memory (‘active cells per pattern’) decreased, the memory capacity of the CA1-PC network increased, so interference effects between stored patterns decreased, and mean recall quality increased. An increase in network size (from 100 to 300 PCs) had no effect on the performance of the three models tested. Another key finding of our study was that increased firing frequency response of a presynaptic inhibitory cell (BSC) inhibiting a network of PCs had a better success at removing spurious activity at the network level and thus improving recall quality than an increased synaptic efficacy of a presynaptic inhibitory cell (BSC) on a postsynaptic PC while keeping its presynaptic firing rate fixed.

## Data Availability

The source code of the model and data are available upon request to the corresponding author. The mathematically formalism of the model can be found in the supplementary online materials of [29].
